# Tissue-resident Sca1+ PDGFRα+ mesenchymal progenitors are the cellular source of fibrofatty infiltration in arrhythmogenic cardiomyopathy

**DOI:** 10.12688/f1000research.2-141.v1

**Published:** 2013-06-19

**Authors:** Ben Paylor, Justin Fernandes, Bruce McManus, Fabio Rossi

**Affiliations:** 1 Biomedical Research Center, University of British Columbia, Vancouver , V6T 1Z3, Canada; 2 James Hogg Research Centre, University of British Columbia, Vancouver, V6Z 1Y6, Canada

## Abstract

Arrhythmogenic cardiomyopathy (AC) is a disease of the heart involving myocardial dystrophy leading to fibrofatty scarring of the myocardium and is associated with an increased risk of both ventricular arrhythmias and sudden cardiac death. It often affects the right ventricle but may also involve the left. Although there has been significant progress in understanding the role of underlying desmosomal genetic defects in AC, there is still a lack of data regarding the cellular processes involved in its progression. The development of cardiac fibrofatty scarring is known to be a principal pathological process associated with ventricular arrhythmias, and it is vital that we elucidate the role of various cell populations involved in the disease if targeted therapeutics are to be developed. The known role of mesenchymal progenitor cells in the reparative process of both the heart and skeletal muscle has provided inspiration for the identification of the cellular basis of fibrofatty infiltration in AC. Here we hypothesize that reparative processes triggered by myocardial degeneration lead to the differentiation of tissue-resident Sca1+ PDGFRα+ mesenchymal progenitors into adipocytes and fibroblasts, which compose the fibrofatty lesions characteristic of AC.

## Introduction

Arrhythmogenic cardiomyopathy (AC) is a heterogeneous disease of the heart associated with an increased risk of both ventricular arrhythmias and sudden cardiac death. Although the exact pathogenesis of this disease is unknown, mutations in genes coding for the five major proteins of the desmosome, namely plakoglobin, desmoplakin, plakophilin-2, desmoglein ^1^ and desmocollin-2 ^2^, have been strongly implicated. The penetrance of these desmosomal mutations in AC patients has been estimated to be between 40 and 90% by different studies ^3– 5^, making identification of at-risk individuals by clinical genotyping difficult. As such, there is currently no single test to diagnose AC, although improved quantitative functional parameters associated with the disease as well as identification of pathogenic mutations in first-degree relatives have improved both the sensitivity and specificity of current diagnostic criteria ^6^. Current therapeutic and management paradigms rely on symptomatic impact including anti-arrhythmic drugs and lifestyle modifications, and it is hoped that probing the link between desmosomal gene defects and the progression of AC will lead to more effective disease-targeted therapies ^7^.

The name, arrhythmogenic cardiomyopathy, has gradually evolved since the initial classification of this disease in 1982 when it was called "right ventricular dysplasia" ^8^ as it was thought to be caused by a developmental defect of the heart before birth. It was soon determined that the symptoms and signs were in fact a progressive cardiac disease, and thus dysplasia was replaced with "cardiomyopathy" ^9^. Clinically, since the main symptoms that appear during the progression of the disease are right ventricular arrhythmias, the term arrhythmogenic right ventricular cardiomyopathy (ARVC) became the most commonly used name. With a growing body of evidence demonstrating left ventricular involvement in this disease ^10, 11^, the current terminology of arrhythmogenic cardiomyopathy (AC) was adopted in 2010 ^7^. Several disease patterns are encompassed by this definition; including right dominant, left dominant and biventricular AC ^6, 7^.

## Pathophysiological mechanisms of arrhythmogenic cardiomyopathy

While several etiopathogenic theories for the development of AC have been proposed ^12, 13^, including dysontogenic (dysplasia) ^14^, apoptotic ^15, 16^ and transdifferentiative ^17^ processes, it is most widely accepted today that degenerative and dystrophic mechanisms underlie its progression ^7^. This latter model, proposed well before the discovery of associated desmosomal-mutations, draws from histopathological similarities between AC and skeletal-muscular dystrophies, which are both characterized by progressive muscle damage with associated replacement with fibrofatty connective tissue. Experimental data have demonstrated that cardiomyocyte death, either by apoptosis or necrosis, is the primary initiating trigger that eventually is followed by fibrofatty replacement of functional myocardium ^18, 19^. The molecular pathways that underlie the progressive loss of cardiomyocytes in AC continue to be investigated (reviewed in Shirokova and Niggli (2012) ^20^), with the ultimate goal of developing targeted preventives or therapies. It should be noted that while the presence of fibrofatty tissue is pathognomonic for this disease, Burke *et al.* ^21^ suggest that the fibrofatty infiltration is most likely secondary to associated desmosomal gene mutations. Progressive loss of cardiomyocytes due to mutated desmosomal gene products may activate reparative processes and lead to progressive accumulation of diffuse fibrofatty tissue, with concomitant alterations in cardiac electrophysiological and contractile function. Although it is widely accepted that a key pathological link between desmosomal mutations and the often fatal ventricular arrhythmias of this disease is the development of the characteristic fibrofatty infiltrate, the cellular source of the fibroblasts and adipocytes that compose this connective tissue currently remains unclear. Two recent studies in murine models of the disease have implicated cardiac progenitor cell (CPC) populations as the most likely candidate, but noted that further work was necessary to clearly identify the cells involved ^22, 23^.

## The hypothesis

We propose that reparative processes in the heart triggered by myocardial dystrophy lead to the differentiation of tissue-resident cardiac Sca-1+ PDGFRα+ mesenchymal progenitors into both fibroblasts and adipocytes, resulting in the characteristic fibrofatty lesion observed in AC.

## Cellular source of fibrofatty infiltration in arrhythmogenic cardiomyopathy

Recent human genetic analyses of AC patients ^24^ have identified the causative role of desmosomal mutations in its progression and have aided in the clinical diagnosis of the disease, but it has been the development of numerous *in vivo* transgenic murine models of AC ^25^ that have greatly furthered our fundamental understanding of the disease.

Utilizing lineage tracing and genetic fate mapping, Lombardi *et al.* showed recently that second heart field CPCs are a source of adipocytes ^23^ in a murine model of AC. Through use of a series of conditionally expressed reporter strains, which concomitantly delete the desmosomal protein desmoplakin in cardiac myocyte lineages and permanently activate yellow fluorescent protein expression in the deleted cells, this group elegantly demonstrated a contribution of second heart field CPCs to adipogenesis. Further, they provided strong evidence implicating perturbations in Wnt/Tcf712 signaling as a molecular mechanism underlying this progression. Although these experiments have provided compelling data to support the molecular mechanisms governing the development of fibrofatty scar tissue in AC, other than demonstrating the involvement of Isl-1+ second heart field progenitors, the lineage tracing strategies were unable to provide substantive evidence as to the identity of cells involved in fibrofatty scar development. Further, they were unable to conclude that the second heart field CPCs are the sole cellular source since the Cre-drivers used (Nkx2.5, Mef2C, α-MyHC) are unable to distinguish the involvement of pericytes, fibroblasts or circulating cells.

It was demonstrated long ago that CPCs identified using the marker Sca-1 can give rise to adipocytes *in vitro* ^26^, yet evidence implicating Sca-1+ CPCs as the cellular source of adipocytes in models of AC *in vivo* is still lacking. Lombardi *et al.* ^22^ have identified a role for Sca-1+ CPCs in the enhanced adipogenesis of AC mice harbouring mutations in the desmosomal protein plakoglobin. They did not, however, perform the strict lineage tracing experiments required to quantitatively assess the relative contribution of CPCs to the adipogenesis in their models. The long standing dogma of AC being primarily a right ventricular disease was supported by demonstration of the involvement of second-heart field progenitors, but this proposed model of AC fails to accommodate recent reports of similar pathological processes in the left ventricle ^17, 27^. Indeed, data demonstrating that fibrofatty scarring and functional deterioration occur in both ventricles in AC argues that the progenitor cell subset responsible for generation of fibroblasts and adipocytes in this disease is distributed throughout the heart. If such is true, a population of subepicardial progenitors may be a good candidate ^28^. Such a notion is strongly supported by recent studies demonstrating that both the murine ^29^ and human ^30^ heart harbour a population of pro-epicardially-derived tissue-resident mesenchymal progenitors, which express Sca1 and PDGFRα. These studies demonstrated the broad trans-germ layer differentiative capacity of this cardiac-resident progenitor population ^29^ and characterized their presence in both fetal and diseased human myocardium ^30^, but did not thoroughly investigate their role in regeneration and repair of the heart. The notion that the heart harbours a population of Sca1+ PDGFRα+ mesenchymal progenitors able to generate both fibroblasts and adipocytes aligns with the hypothesis that this population is the most significant cellular source of fibrofatty infiltration in AC.

Although fibrofatty replacement of the myocardium is the hallmark feature of AC, the consistent observation of small lymphocytic foci in the disease ^31– 33^ further supports the involvement of cardiac-resident Sca1+ PDGFRα+ mesenchymal progenitors in its progression, as these progenitors have been recently shown to contribute *in vivo* to lymph node stroma ^34^ and follicular dendritic cells ^35^. Thus, the milieu of chronic myocardial inflammation may trigger their differentiation not just into adipocytes and myofibroblasts, but also possibly in cells capable of attracting and supporting lymphocytes.

Although studies in murine models of AC employing the strict lineage tracing methods required to determine the cellular source of adipocytes have yet to be performed, studies in skeletal muscle (SM) further support our hypothesis in implicating Sca1+ PDGFRα+ tissue-resident mesenchymal progenitors. Recent studies ^36, 37^ accomplish the prospective isolation and purification of a population of SM resident Sca1+ PDGFRα+ mesenchymal progenitors, which were further shown to be the sole SM-derived population with fibro-adipogenic potential. Additional evidence from *mdx* mice, a murine model of Duchenne muscular dystrophy ^38^, strongly supports the notion that tissue-resident Sca1+ PDGFRα+ cells are the principal cell population involved in the generation of fibrofatty scars in situations of chronic muscle damage. Interestingly, a robust regenerative capacity of SM was demonstrated by these studies, and others ^39, 40^, to be at least partly due to paracrine roles of these tissue-resident mesenchymal progenitors. In light of this evidence, the therapeutic potential of modulating proliferation and differentiation of cardiac Sca1+ PDGFRα+ progenitor cells in situations of acute or chronic damage is certainly intriguing. With a large body of clinical evidence demonstrating the beneficial effects of transplanting bone-marrow derived mesenchymal stromal cells (MSCs) into patients afflicted with a variety of cardiovascular disorders ^41^, and numerous data highlighting the similarities between MSCs derived from different tissues ^42^, the identification of a tissue-resident counterpart presents an attractive candidate for pharmacological modulation. Such manipulation could lead to therapeutic benefits, similar to those observed with exogenous delivery of similar cells but devoid of the adversities stemming from *ex vivo* manipulations, including (but not limited to) transplantation, immunogenicity issues and challenges involved in the use of good manufacturing practice facilities for clinical-grade cell preparations.

## Research required to evaluate the hypothesis

In order to unequivocally demonstrate the contribution of cardiac-resident Sca1+ PDGFRα+ mesenchymal progenitors to fibrofatty scarring in AC, a number of questions must be addressed.

First, a more thorough characterization of these progenitor cells must be performed to determine both their functional role in health and disease, as well as determine the homogeneity of this population. It is very possible that within this phenotypic identity, several functionally different cellular subsets are present, and the unravelling of these hierarchies could provide significant insight into cellular processes in the regenerating or degenerating myocardium. Dularoy *et al.* ^43^ identified a fibrogenic subpopulation within Sca1+ PDGFRα+ progenitor in the SM using ADAM12+, highlighting heterogeneity, and supporting the need to further distinguish between functional subsets of this mesenchymal population. It will also be highly important to determine whether the desmosome plays a direct role in Sca1+ PDGFRα+ progenitor cell function, or whether the arrhythmogenic reparative disorder observed in AC is to be ascribed solely to continued loss of cardiomyocytes due to desmosomal gene defects.

Second, further lineage tracing experiments using several inducible Cre-drivers such as PDGFRα-CreER in combination with either previously described ^25^ or novel models of AC will allow identification of the role of this population in AC.

Finally, the limitations of the murine Sca-1 (Ly6A/E) as a marker of tissue-resident progenitors should be addressed since there is currently no known human homolog for this gene. Such is the case despite the hypothesis that a broad range of functions mediated by this marker are probably assumed by other Ly6 proteins in humans ^44^.

## Consequences of the hypothesis

With no current treatment available for AC, it is highly attractive to consider that manipulation of molecular mechanisms underlying the development of the cardiac fibrofatty infiltration could mitigate both functional deterioration as well as survival of AC patients. Clinical trials in patients suffering from Duchenne muscular dystrophy utilizing anti-fibrotic strategies ( NCT01521546) may provide valuable insight into the beneficial effects of preventing development of fibrofatty scars in the functional myocardium ^45^. Additionally, with several recent reports describing *in vivo* reprogramming of cardiac fibroblasts into functional cardiomyocytes ^46– 48^, it could be postulated that targeting of existing fibrofatty scars could be therapeutically beneficial. With a growing recognition of functional and theoretical overlap between fibroblasts and mesenchymal stem cells ^49, 50^ there is a strong possibility that the beneficial effects seen in recent cardiac studies are due to reprogramming of these tissue-resident progenitor cells. Recent technical advances involving patient-specific induced pluripotent stem cells (iPSCs) ^51^ as well as further development of aforementioned reprogramming strategies may enable novel targeted approaches to specific cell populations with enhanced transdifferentiative capacities, and could provide a basis for next-generation therapy of AC.

## Conclusion

In summary, recent evidence demonstrating that the heart harbours a population of Sca-1+ PDGFRα+ mesenchymal progenitors provides new directions for defining the cellular source of the fibrofatty infiltrate that is characteristic of AC. A growing understanding of the role of tissue-resident mesenchymal progenitors in numerous other tissues, most notably SM, offers strong support for our hypothesis. Potential avenues for novel targeted therapeutics may emerge to benefit patients with AC.
