# Supplementary material for: Tissue-resident Sca1+ PDGFRα+ mesenchymal progenitors are the cellular source of fibrofatty infiltration in arrhythmogenic cardiomyopathy
Source: F1000Res. 2013 Jun 19;2:141. [Version 1] doi: 10.12688/f1000research.2-141.v1 (PMC3790611; doi:10.12688/f1000research.2-141.v1)
Supplement: Peer review history

# Peer review: Referee response for version 1

**Published:** 2013-06-20

**Authors:** Richard Harvey

**Affiliations:** 1 Victor Chang Cardiac Research Institute, Darlinghurst, Australia

This is an excellent synthesis of the literature surrounding the history, clinical manifestations, genetic basis and aetiology of arrhythmogenic cardiomyopathy (AC), and in particular, proposes a role for the resident Sca1+ PDGFRα+ interstitial population that includes mesenchymal (MSC-like) progenitor cells in the fibrofatty infiltrates that develop during the disease. This hypothesis piece notes carefully the limitations of conclusions arising from published studies using animal models that hint at a role for second heart field cardiomyocyte progenitors in the development of disease. Of importance, is whether the fibrofatty infiltrates are the primary or secondary target of the mutations in desmosomal component genes that underpin the disease. The piece is scholarly, and well written. A dimension not explored extensively is the possibility that the fibrofatty cells arise from the epicardium of the adult heart, which has been shown by a number of groups to become activated after injury. Activated epicardium re-expressed genes involved in the fetal epicardial program and reveals a latent lineage reserve that can contribute fibroblasts and smooth muscle cells to the infarct zone of a chronically injured ischaemic heart. While the Sca1+ PDGFRα+ interstitial mesenchymal cells and epicardial cells share a lineage relationship in development, an additional challenge in the lineage mapping studies proposed is to distinguish whether fibrofatty infiltrates in AC arise from one or the other, or both, of these cellular compartments. Nonetheless, the ideas explored in this article overlap with interesting and important issues in cardiac developmental and stem cell biology, and with the potential for targeting fibrofatty progenitor cells as a therapy in arresting the progression of AC in the clinical setting. An excellent work that should be a reference point for future studies.

I have read this submission. I believe that I have an appropriate level of expertise to confirm that it is of an acceptable scientific standard.

# Peer review: Referee response for version 1

**Published:** 2013-06-20

**Authors:** James Chong

**Affiliations:** 1 Westmead Millennium Institute for Medical Research, Westmead, Australia

This is a considered hypothesis for cellular origins of the fibro-fatty myocardial scarring underlying the poorly understood clinical disease - Arrhythmogenic Cardiomyopathy. The authors are a group with considerable experience investigating fibroadipogenic and skeletal muscle progenitor cells. 

The points below address the questions posed to reviewers:

- The title is perhaps too strongly worded. Whilst the hypothesis is sound and intriguing, there is currently little or no data proving that the title/hypothesis is true. This is appropriately discussed in the manuscript.

- The abstract does represent a suitable summary of the work.

- The article content provides a thorough review of data supporting the proposed hypothesis. If the hypothesis were proven true this would be a substantial advance for this field and for clinical cardiology

- The conclusions are sensible and justified.

I have read this submission. I believe that I have an appropriate level of expertise to confirm that it is of an acceptable scientific standard.
